# Impaired Autophagy Contributes to Adverse Cardiac Remodeling in Acute Myocardial Infarction

**DOI:** 10.1371/journal.pone.0112891

**Published:** 2014-11-19

**Authors:** Xiaoqian Wu, Lishan He, Fajiang Chen, Xiaoen He, Yi Cai, Guiping Zhang, Quan Yi, Meixiang He, Jiandong Luo

**Affiliations:** 1 Department of Pharmacology, Guangzhou Medical University, Guangzhou, PR China; 2 Guangzhou Institute of Cardiovascular Disease, Guangzhou Key Laboratory of Cardiovascular Disease, and the Second Affiliated Hospital, Guangzhou Medical University, Guangzhou, PR China; University of Udine, Italy

## Abstract

**Objective:**

Autophagy is activated in ischemic heart diseases, but its dynamics and functional roles remain unclear and controversial. In this study, we investigated the dynamics and role of autophagy and the mechanism(s), if any, during postinfarction cardiac remodeling.

**Methods and results:**

Acute myocardial infarction (AMI) was induced by ligating left anterior descending (LAD) coronary artery. Autophagy was found to be induced sharply 12–24 hours after surgery by testing LC3 modification and Electron microscopy. P62 degradation in the infarct border zone was increased from day 0.5 to day 3, and however, decreased from day 5 until day 21 after LAD ligation. These results indicated that autophagy was induced in the acute phase of AMI, and however, impaired in the latter phase of AMI. To investigate the significance of the impaired autophagy in the latter phase of AMI, we treated the mice with Rapamycin (an autophagy enhancer, 2.0 mg/kg/day) or 3-methyladenine (3MA, an autophagy inhibitor, 15 mg/kg/day) one day after LAD ligation until the end of experiment. The results showed that Rapamycin attenuated, while 3MA exacerbated, postinfarction cardiac remodeling and dysfunction respectively. In addition, Rapamycin protected the H9C2 cells against oxygen glucose deprivation *in vitro*. Specifically, we found that Rapamycin attenuated NFκB activation after LAD ligation. And the inflammatory response in the acute stage of AMI was significantly restrained with Rapamycin treatment. *In vitro*, inhibition of NFκB restored autophagy in a negative reflex.

**Conclusion:**

Sustained myocardial ischemia impairs cardiomyocyte autophagy, which is an essential mechanism that protects against adverse cardiac remodeling. Augmenting autophagy could be a therapeutic strategy for acute myocardial infarction.

## Introduction

Acute myocardial infarction (AMI) is one of the most common heart diseases that cause morbidity and mortality worldwide [1]. Following AMI, heart failure with adverse remodeling of the left ventricle (LV) characterized by cavity dilatation and diminished cardiac performance is the most common outcome. The process of cardiac remodeling is complicated, however, many other factors, including death or hypertrophy of cardiomyocytes, inflammation and fibrosis within the infarct tissue, are all associated with disease progression during the chronic stage [2], [3]. Therefore, understanding the mechanisms underlying post-AMI heart remodeling is essential for effective retardation against AMI.

Autophagy, a highly conserved energy-dependent process implicated as a cell survival/death mechanism, degrades and recycles organelles and long-lived proteins to maintain cellular homeostasis and adaptation to nutrient depletion [4]. The constitutive autophagy in the heart under baseline is a homeostatic mechanism for maintaining cardiac structure and function, and upregulation of autophagy in the failing hearts protected cardiomyocytes from pressure overload [5]. Autophagy is well elucidated as a key regulator of ischemia/reperfusion injury and it was believed that autophagy played distinct roles in the heart during ischemia and reperfusion [6], [7], [8]. The induction of autophagy in the ischemia phase is protective, whereas reperfusion-stimulated autophagy is implicated in cardiomyocyte death due to the impaired autophagosome clearance [6], [7]. However, the dynamics and functional roles of autophagy in a longer term of myocardial ischemia remain unclear and controversial. Autophagy was shown as an adaptive response of the heart that protected the myocardium from acute ischemic death [9], [10]. Whereas it was reported that autophagy may result in cardiomyocyte death which exacerbates heart failure [11], [12]. The dynamics and functional role of autophagy in the sustained myocardial ischemia are poorly understood.

Our aim in the present study was to investigate the dynamics and role of autophagy and the mechanism(s), if any, in the progression of AMI. To address this issue, we used a mouse acute myocardial infarction model *in vivo* and H9C2 cells oxygen glucose deprivation model *in vitro*, first observing the dynamics of autophagy in the hearts of AMI and then examining the effects of autophagy on the progression of postinfarction cardiac remodeling and dysfunction.

## Materials and Methods

### Animals and Experimental Protocols

This study was carried out in strict accordance with the recommendations in the Guide for the Care and Use of Laboratory Animals published by the United States National Institutes of Health (NIH publication no. 85-23, revised 1996). The protocol was approved by the Animal Research Committee, Guangzhou Medical University, Guangzhou, China. Adult male C57/B6 mice (body weight around 22∼25 g, aging between 8 and 10 weeks) were obtained from Medical Experimental Animal Center of Guangdong Province (Guangzhou China) and received humane care. Myocardial infarction was generated in the male C57BL/6J mice by ligating the LAD coronary artery as we have previously described [13]. Briefly, mice were anesthetized with sodium pentobarbital (50 mg/kg) intraperitoneally and artificially ventilated with a respirator, and all efforts were made to minimize suffering. Myocardial infarction was followed by making a slipknot (8-0 silk) around the left anterior descending coronary artery. Sham-treated animals underwent the same surgical procedure without ligating LAD coronary artery.

Mice were randomized into 4 groups: (i) sham group (Sham, *n* = 46); (ii) AMI + vehicle group, AMI mice received the same volume of saline alone (CTL, *n* = 41); (iii) AMI + Rapamycin, AMI mice received Rapamycin (Rapa, *n* = 41); (iv) AMI + 3MA, AMI mice received 3-Methyladenine (3MA, *n* = 41). Rapamycin (2 mg/kg/day [14]; Cat#R-5000, LC laboratories, New Boston, USA) or 3MA (15 mg/kg/day [15]; Cat#M9281, Sigma-Aldrich, St. Louis, MO, USA) was injected intraperitoneally one day after induction of AMI, and the dose was given once a day and continued for 7 or 21 days.

### Echocardiography

As we have described previously [13], transthoracic echocardiography was performed with a VisualSonics (Vevo 2100; VisualSonics Inc., Ontario, Canada) equipped with a 30 MHz imaging transducer on day 7 and day 21 before sacrificing the mice respectively. Mice were kept anaesthetized with 2% isoflurane gas with an inflow rate of 0.5–1.5 ml/min during the echocardiographic examination. The left ventricle (LV) was analyzed through the parasternal long- and short-axis views. Throughout the procedure, ECG, respiratory rate (RR), and heart rate (HR) were monitored. The body temperature of mice was monitored using a rectal thermometer and was maintained between 36 and 38***°***C. The heart rate was maintained between 350 and 450 beats/min. After measurement, the cardiac output values such as EF (ejection fraction), FS (fractional shortening), LVIDD (LV internal diameter at end-diastole) and LVIDS (LV internal diameter at end-systolic) were calculated according to the guidelines accompanying the Vevo 2100.

### Determination of infarct size

After determination of the cardiac function on day 7 and day 21 respectively, mice were anaesthetized with sodium pentobarbital (100 mg/kg) and assessed to be fully anaesthetized and sacrificed. The ventricles were collected and sliced transversely into 2-mm-thick slices. The slices were incubated in 1% 2, 3, 5-triphenyl tetrazolium chloride (TTC; pH 7.4) for 20 min at 37°C. The infarct area was shown as the area unstained by TTC and was measured by Image-Pro plus 5.0 (Media Cybernetics Inc., MD, USA). Infarct size was expressed as a percentage of left ventricular volume (% = infarct size/left ventricle area) as we have described previously [16].

### Masson’s trichrome staining

After determination of the cardiac function on day 7 and day 21 respectively, mice were anaesthetized and sacrificed as described in section 2.3. The hearts were perfused, sectioned and then fixed in paraffin and subjected to Masson’s trichrome-staining as we have described previously [13].

### Immunohistochemistry

By the end of the experiment, mice were anaesthetized and sacrificed as described in section 2.3. The hearts were collected and fixed in 10% buffered formalin, embedded in paraffin, cut into 4-µm-thick sections. Immunohistochemistry was performed as we have described previously [13] using the following primary antibodies: rabbit polyclonal anti-IL-1β (1∶50 dilution; Cat#16806-1-AP, Proteintech, USA), rabbit polyclonal anti-TNF-α (1∶50 dilution; Cat#BS6000, Bioworld Technology, USA) and rabbit polyclonal anti-IL-6 (1∶100 dilution; Cat#21865-1-AP, Proteintech, USA), mouse monoclonal anti-CD68 (1∶200 dilution in; Cat#ab955, Abcam, Cambridge, USA) and rat monoclonal anti-CD45 (1∶100 dilution; Cat#05-1416, Millipore, MIT, USA).

### Electron microscopy

As we have described previously [16], fractions (1 mm^3^) of the border zone of the ischemic heart were pre-fixed in a solution of 2.5% glutaraldehyde and 1% osmium tetroxide, post-fixed in 1% OsO_4_, dehydrated in an ascending series of alcohols, and embedded in epoxy resin. Ultrathin sections were stained with uranyl acetate and lead citrate. Samples were viewed under a transmission electron microscope (HITACHI H-600, Japan).

### H9C2 cell culture and oxygen-glucose deprivation (OGD)

The H9C2 cells, a subclone of the original clonal cell line derived from embryonic BD1X rat heart tissue, were purchased from the American Type Culture Collection (ATCC, Rockville, MD). The cells were cultured as monolayer in Dulbecco’s Modified Eagle’s Medium (DMEM, Cat#11995-065, Life technologies, USA) containing 4 mM L-glutamine, 4.5 g/L glucose, 10% (v/v) fetal bovine serum (Cat#10082-147, Gibco, USA). All the cells were grown under an atmosphere of 5% (v/v) CO_2_ in air at 37°C. Incubation of Rapamycin (1∼1000 nM), 3MA (10 mM) or Chloroquine (20 uM, Cat#C6628, Sigma) was 2 hours before OGD treatment. Incubation of PDTC (100 uM, Sigma) was 1 hour before OGD. H9C2 cells for OGD treatment were rinsed twice with serum-free, glucose and sodium pyruvate free DMEM (Cat#D5030, Sigma) and were cultured in the same medium at 37°C in a anoxia chamber (InVivo 500; Ruskinn Life Science) saturated with 94%N_2_/5%CO_2_/1%O_2_ for different time as indicated [17].

### RNA interference

siRNA for downregulating gene expression was done by transfection of RNA oligonucleotides with lipofectamine 2000 (Cat#11668019, Life technologies, USA) according to the manufacturer’s instructions. The negative control (NC) siRNA and siRNAs against *ATG5* were synthesized by Ribobio (Guangzhou, China). For *ATG5*, two siRNA oligonucleotides were used.

ATG5-1∶5′-GGCAUUAUCCAAUUGGCCUACUGUU-3′, ATG5-2∶5′-AGGCUCACUUUAUGUCAUGUGUGAA-3′.

### MTT assay

H9C2 cells were seeded in 96-well plates (Corning, USA) at a density of 1×10^3^ cells per well with 100 µl medium containing 10% FBS and incubated at 37°C with 5% CO_2_. H9C2 cells were pretreated with Rapamycin (1, 10, 100, 1000 nM), 3MA (10 mM) or si*ATG5* (100 nM) for 2 hours and then with OGD treatment for different time points were indicated. The growth rate of cells was examined with Cell Proliferation Kit I according to manufacturer's instruction (Kaiji, Nanjing, China). The absorbance of the purple solution was determined at 570 nm wavelengths with a microtiter plate reader (Bio-Rad, USA). Cell viability was calculated as follows: MTT metabolic rates (%) = A570 in experimental well/A570 in control well.

### Trypan blue staining

The trypan blue staining method was used to assess the cell viability. H9C2 cells were seeded in 96-well plates at a density of 1×10^3^ cells per well with 100 µl medium containing 10% FBS and incubated at 37°C with 5% CO_2_. Different concentration of Rapamycin (1, 10, 100, 1000 nM) was pretreated for 2 hours before OGD for 24 hours. The cells were mixed with 0.4% Trypan blue solution (Sigma, USA). The viable cells with intact cell membranes, which could exclude the dye, were counted using a hemocytometer (Marienfeld, Germany) and expressed as percentage of the total cells counted (cell viability).

### TUNEL assay

DNA fragmentation was visualized by use of the ApopTag kit (Roche, Switzerland). This system labels free 3′OH termini of DNA in cells with digoxigenin-tagged nucleotides with the use of the enzyme terminal deoxynucleotidyl transferase. Total nuclei were stained by 4′,6′-diamidino-2-phenylindole (DAPI, Bioss, Beijing, China). The H9C2 cells were grown on 48-well plates and pretreated with Rapamycin (100 nM) for 2 hours before OGD for 12 hours or 24 hours. Cells only labeled as being TUNEL positive were expressed as percentage of the total nuclei.

### p65 nuclear translocation

The H9C2 cells were grown on 48-well plates and pretreated with Rapamycin (100 nM) for 2 hours before OGD for 1 hour. Cells were fixed with 4% paraformaldehyde in phosphate-buffered saline for 30 minutes. After blocking for 2 hours with 1% bovine serum albumin, the cells were incubated with rabbit anti-p65 (1∶1000, Cat#9936S, Cell Signaling Technology) at 4°C overnight. After washing with PBS, dylight549-confugated secondary antibody (1∶500, Cell signaling technology) was added for 1 hour in the dark. Nuclei were stained with DAPI, and the cells were visualized under fluorescence microscope.

### pEGFP-LC3 plasmid transfection

Autophagy was assayed by fluorescence microscope quantification of fluorescent autophagosomes in H9C2 cells. Cells were transfected with 1.4 µg pEGFP-LC3 and 4 µL Lipofectamine 2000 for 4–6 hours and then cultured in DMEM with 10% fetal calf serum for 24 hours before OGD. The percentage of pEGFP-LC3 positive cells with pEGFP-LC3 puncta was assessed by counting a minimum of 100 cells for duplicate samples per condition in three independent experiments, while the number of pEGPF-LC3 puncta per pEGFP-LC3 positive cell was assessed by counting a minimum of 50 cells for duplicate samples per condition in three independent experiments.

### Dual-Luciferase Reporter Assay

As we have described previously [18], the H9C2 cells were plated in 48-well plates 24 hours prior to transfection. PGL4.32-NF-kB-RE-luc2P and pRL-TK vector (0.2 µg DNA/each) as the control was transiently transfected by using 1 µL Lipofectamine 2000. 24 hours after transfection, cells were treated with 100 nM Rapamycin for 2 hours before OGD for 1 hour, and then firefly renilla luciferase activities were measured with a Luminoskan Ascent luminometer (Promega, USA). Results were expressed as fold change in the ratio of luciferase activity (RLA) of the PGL4.32-NF-kB-RE-luc2P vector compared with that of the RLA of PRL-TK vector.

### ELISA

As we have described previously [19], the tissue homogenates from the infarct border zone of 1 day and 7 day after AMI were processed. TNF-α, IL-1β and IL-6 were measured by ELISA kits (Cat#EMC102a, Cat#EMC001b and Cat#EMC004, NeoBioscience, Shenzhen, China). The reaction mixture was read using the multifunction protein array reader (Thermo, USA) and data was analyzed with GraphPad Prism 5.0.

### Western blot analysis

Western blot analysis was performed as we described previously [18]. The border zone of the infarct hearts was separated and homogenized. Equal amounts of protein (30 mg) were separated on SDS–polyacrylamide gels (10%) and electrotransferred to polyvinylidene difluoride membranes (Roche, Switzerland). The membranes were incubated with rabbit anti-LC3 (1∶1000 dilution; Cat#L7543, Sigma), rabbit anti-Beclin-1 (1∶1000 dilution; Cat#3738, Cell signaling technology), rabbit anti-p65 (1∶1000 dilution; Cat#9936S, Cell signaling technology), mouse anti-IκBα (1∶1000 dilution; Cat#9936S, Cell signaling technology), rabbit anti-P62(Cat#5114 Cell signaling technology), rabbit anti-AMPK (Cat#5831, Cell signaling technology), rabbit anti-Phospho-AMPK (Cat#2535, Cell signaling technology), mouse anti-HIF-1α (NB100-105, Novus Biologicals), goat anti-GAPDH and goat anti-β-actin (1∶1000 dilution; Cat#SC-48166 and Cat#SC-1616, Santa Cruz Biotechnology) at 4°C overnight, and incubated with either goat anti-rabbit, rabbit anti-mouse or mouse anti-goat second antibody (1∶5000 dilution; Santa Cruz Biotechnology) for 1 hour at room temperature. Blots were developed using a chemiluminescent substrate and molecular band intensity was determined by densitometry.

### Statistical analyses

All data were analyzed with the statistical software GraphPad Prism 5.0 (GraphPad Software Inc. La Jolla, CA), and were expressed as means ± SEM. The differences between two groups were analyzed using Student’s unpaired t-test, and differences between three or more groups were evaluated via one-way ANOVA with Bonferroni correction. *P*<0.05 was considered statistically significant.

## Results

### Autophagy was induced sharply and however impaired after sustained myocardial ischemia *in vivo* and *in vitro*

**Figure 1:**
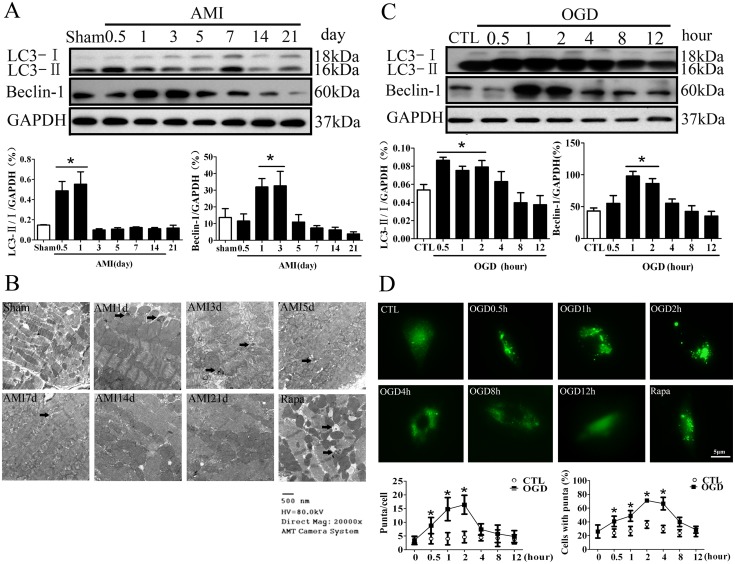
Autophagy is induced sharply and however impaired after myocardial ischemia stimuli *in vivo* and *in vitro*. **A**, autophagy-associated protein marker LC3 and Beclin 1 were examined by Western blotting in the infarct border zone of different time points after LAD ligation (n = 5, *P<0.05 *vs* Sham). **B**, The representative images of the electron microscopic analysis of the infarct border zone of different time points after LAD ligation. Rapamycin (2 mg/kg/day) was administered intraperitoneally for 21 days as a positive control. The arrows indicated autophagosomes. **C**, The protein expression of LC3 and Beclin 1 was examined in the H9C2 cells at different time points of OGD. (n = 8, *P<0.05 *vs* CTL). **D**, GFP-LC3 modification in the H9C2 cells at different time points of OGD (n = 5, *P<0.05 *vs* CTL). The H9C2 cells were treated with Rapamycin (100 nM) for 2 hours as a positive control. AMI, acute myocardial infarction; OGD, oxygen glucose deprivation; CTL, normal control.

To explore the role of autophagy in the ischemic heart diseases, it is important to understand how autophagy is regulated in the hearts under the ischemia stimuli. Mice were subjected to AMI by ligating left anterior descending coronary artery permanently. The protein marker for autophagy, LC3 modification in the infarct border zone was increased sharply 12 to 24 hours after LAD ligation, and however, decreased from day 3 after LAD ligation and kept a low level throughout the latter phase of AMI (Figure 1A, *P*<0.05 *vs* Sham, n = 6). Another autophagy maker protein, Beclin 1 expression exhibited the similar tendency. Notably, the electron microscopic analysis of the infarct border zone revealed the presence of cytoplasmic vacuoles that resembled autophagosomes and often contained intracellular organelles, such as degraded mitochondria and membrane-like structures, autophagic vacuoles in the hearts of day 1 after LAD ligation. The typical autophagosomes with smaller size became more numerous in the hearts of day 3 and day 5 after LAD ligation. There were autophagic vacuoles in the infarct border zone after Rapamycin treatment for 21 days (Figure 1B). We further examined autophagic flux by testing P62 degradation with Western blotting. P62 degradation was increased from day 0.5 to day 3, and however, decreased from day 5 until day 21 after LAD ligation (Figure S1A). Consequently, these results indicated that autophagic flux was induced in the acute phase of AMI, and however, impaired in the latter phase of AMI.

*In vitro*, we examined the effect of oxygen glucose deprivation on autophagy. We found that LC3 modification and Beclin 1 expression were increased from 0.5 to 2 hours, and then decreased until 12 hours with oxygen glucose deprivation (Figure 1C). When autophagy is induced, the LC3 protein is processed and lipidated, becoming incorporated into the expanding phagophore membrane. GFP-LC3 is therefore frequently used as a marker for autophagy; in particular it translocates from a mainly cytosolic to a punctuate localization upon autophagosome accumulation. The H9C2 cells were treated with Rapamycin (100 nM) for 2 hours as a positive control and showed the typical LC3 puncta (Figure 1D). When we expressed GFP-LC3 in the H9C2 cells, the GFP signal was largely diffuse, with only 26.04% of cells containing any puncta and an average of 3.4 puncta per cell. GFP signal was increased to 40.85% of cells containing between 5 and 11puncta, with an average of 8.8 puncta per cell 30 minutes after OGD treatment. LC3 modification arrived at peak around 2 hours after OGD treatment with about GFP signal in 71.10% of cells and an average of 16.4 puncta per cell. However, LC3 modification decreased even to the base level after 8 hours of OGD treatment (Figure 1D). We further examined P62 degradation in the H9C2 cells after different time of OGD. P62 degradation was increased 2 hours after OGD, and however, P62 was accumulated at 12 hours after OGD (Figure S1B). In addition, we measured P62 degradation to assess the autophagy flux in the H9C2 cells with the lysosomal inhibitor, Chloroquine. The H9C2 cells were treated with Chloroquine (20 uM) for 2 hours before OGD treatment for 12 hours. As shown in Figure S2, OGD treatment resulted in an accumulation of P62 in the H9C2 cells, but Chloroquine did not further change the P62 level after OGD treatment (*P*>0.05, O+CQ *vs* OGD).

Overall, the results of *in vivo* and *in vitro* studies indicated that autophagy was induced dramatically in the acute phase of AMI, and however, impaired in the latter phase of AMI.

### The effect of impaired autophagy in the cardiac function and remodeling after AMI

**Figure 2:**
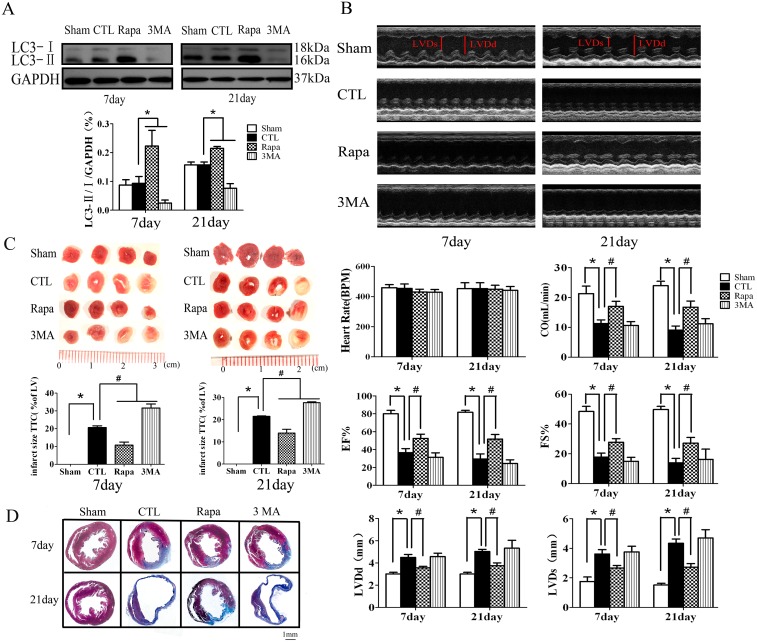
The effect of autophagy in the cardiac function and remodeling after AMI. **A**, Rapamycin induced LC3 modification in the heart tissue (n = 4, *P<0.05 *vs* CTL). **B**, the representative images and analysis results of echocardiographic assessment of hearts subjected to LAD ligation. (n = 8, *P<0.05 *vs* Sham; #*P*<0.05 *vs* CTL). **C**, the representative images and analysis results of TTC staining assessment of the hearts subjected AMI (n = 5, *P<0.05 *vs* Sham; #*P*<0.05 *vs* CTL). **D**, the representative images of Masson’s trichrome staining assessment of the infarct border zone at different time points after LAD ligation. Sham, mice without LAD ligation; CTL, LAD ligation with saline; Rapa, LAD ligation with Rapamycin treatment; 3MA, LAD ligation with 3MA treatment.

Most of the AMI patients in the clinical setting frequently present in the subacute stage. Actually, we are not sure about the effect of the impaired autophagy in the subacute and chronic stages of AMI. To investigate whether enhancing autophagy favorably attenuates myocardial remodeling in the latter phase of AMI, we treated the mice with Rapamycin (an autophagy enhancer, 2.0 mg/kg/day) or 3-methyladenine (3MA, an autophagy inhibitor, 15 mg/kg/day) from one day after LAD ligation for 3 weeks. Rapamycin significantly induced, while 3MA inhibited autophagy in the hearts as shown in Figure 2A. An echocardiography assessment of cardiac function was performed at day 7 and day 21 after LAD ligation. As shown in Figure 2B, compared with sham-operated hearts, the ischemic hearts in the CTL group showed significant reduced cardiac output, fractional shortening and ejection fraction, and enlarged LV end-systolic and LV end-diastolic dimension. Notably, the fractional shortening and ejection fraction were significantly increased in the hearts of 7 day (by 56.05% and 86.79%) and 21 day (by 43.94% and 66.49%) Rapamycin treatment after LAD ligation respectively compared with the hearts of CTL group. Likewise, LV end-systolic and LV end-diastolic dimension were significantly decreased in the hearts of 7 day (by 26.24% and 37.24%) and 21 day (by 20.71% and 25.39%) Rapamycin treatment after LAD ligation respectively compared with CTL. However, the specific autophagy inhibitor, 3MA treatment revealed no obvious effect on the cardiac function compared with the hearts in CTL group.

Further, we performed TTC and Masson’s trichrome staining to examine the involvement of autophagy in the postinfarction cardiac remodeling. As shown in Figure 2C, the infarct size in the mice of Rapamycin treatment after LAD ligation was significantly decreased compared with that of CTL (12.14±4.22% versus 20.67±2.47% for 7 day after LAD, 13.94±4.79% versus 21.50±0.59% for 21 day after LAD, *P*<0.05, n = 6). In contrast, 3MA significantly increased the infarct size compared with that of the CTL group (*P*<0.05, n = 6). Masson’s trichrome staining analysis of the infarct border zone revealed shrunk infarct size and attenuated cardiac fibrosis in the hearts of Rapamycin treatment after LAD ligation compared with that of CTL group, while 3MA worsen the cardiac remodeling compared with CTL (Figure 2D).

Thus, it is suggested that Rapamycin attenuates, while 3MA exacerbates, postinfarction cardiac remodeling and dysfunction respectively.

### The effect of autophagy against oxygen glucose deprivation in the H9C2 cells *in vitro*

**Figure 3:**
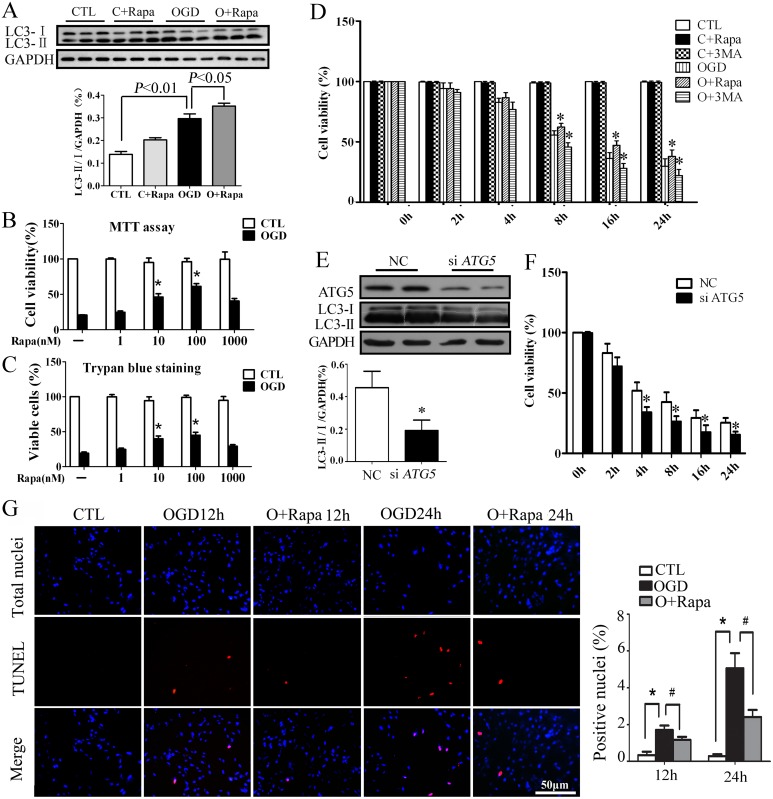
The effect of autophagy against oxygen glucose deprivation in the H9C2 cells *in vitro*. **A**, Rapamycin induced LC3 modification in the H9C2 cells after OGD tested by Western blotting (n = 4). MTT assay (**B**) and trypan blue staining (**C**) to test the effect of different concentrations of Rapamycin on the cell survival after OGD for 24 hours (n = 8, *P<0.05 *vs* OGD without Rapamycin). **D**, MTT assay to test the effect of Rapamycin and 3MA on the cell survival after OGD for different time (n = 8, *P<0.05 *vs* OGD). **E**, The protein expression of ATG5 and LC3 after transfection of si*ATG5* in the H9C2 cells tested by Western blotting (n = 5, *P<0.05 *vs* NC). **F**, The effect of si*ATG5* on the cell survival after OGD for different time in the H9C2 cells tested by MTT assay (n = 8, *P<0.05 *vs* NC). **G**, The representative images and the analysis result of TUNEL staining in the H9C2 cells to test the effect of Rapamycin on cell apoptosis after OGD (n = 6, *P<0.05 *vs* CTL, #*P*<0.05 *vs* OGD). CTL, normal control; OGD, oxygen glucose deprivation; C+Rapa, normal control with Rapamycin treatment; O+Rapa, OGD with Rapamycin treatment. C+3MA, normal control with 3MA treatment; O+3MA, OGD with 3MA treatment. NC, negative control.

To delineate the functional effect of the autophagy in response to anoxia, we treated H9C2 cells with Rapamycin to enhance autophagy and 3MA or si*ATG5* to inhibit autophagy with oxygen glucose deprivation *in vitro*. As shown in Figure 3A and Figure 1D, Rapamycin (100 nM) significantly induced autophagy. Rapamycin (10∼100 nM) significantly improved cell survival with OGD treatment for 24 hours by either MTT assay (Figure 3B) or the trypan blue exclusion method (Figure 3C) compared with OGD without Rapamycin (*P*<0.05, n = 8). Either Rapamycin or 3MA will not affect the cell survival of H9C2 cells under the normal condition. Rapamycin improved cell survival while 3MA reduced cell survival with OGD for the different time points (8 to 24 hours) (Figure 3D). We further transfected the H9C2 cells with *ATG5* siRNA. Transfection of *ATG5* siRNA (100 nM) significantly downregulated the autophagy gene *ATG5* and inhibited autophagy (Figure 3E). As shown in Figure 3F, *ATG5* siRNA reduced the cell survival with OGD, which was consistent with the pharmacological intervention of 3MA. Further, TUNEL staining assay demonstrated that Rapamycin (100 nM) significantly decreased the H9C2 cell apoptosis after oxygen glucose deprivation (Figure 3G).

### Rapamycin attenuated the inflammatory response after acute myocardial infarction

**Figure 4:**
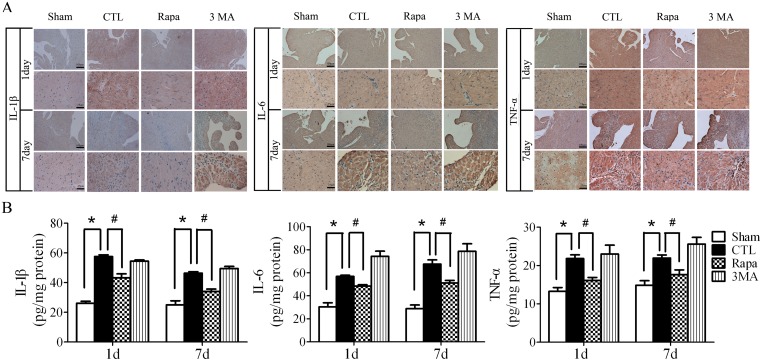
Rapamycin attenuated the inflammatory cytokines production after LAD ligation. **A**, the immunohistochemical assay of the infarct border zone at different time points after LAD ligation. **B**, Elisa assay to quantify the production of the inflammatory cytokines by the hearts after LAD ligation. (n = 5, *P<0.05 *vs* Sham; #*P*<0.05 *vs* CTL). Sham, mice without LAD ligation; CTL, LAD ligation with saline; Rapa, LAD ligation with Rapamycin treatment; 3MA, LAD ligation with 3MA treatment.

To investigate the mechanism by which the enhanced autophagy protected against adverse cardiac remodeling of AMI, we examined the effect of Rapamycin on inflammation which is an important process of AMI. As shown in Figure 4A, the proinflammatory cytokines, IL-1β, IL-6, and TNF-α production in the infarct border zone were significantly increased in the hearts both of day 1 and day 7 after LAD ligation, which was attenuated by Rapamycin treatment. In parallel with the immunohistochemical analysis, Elisa assay showed that Rapamycin treatment restrained the increasing TNF-α, IL-1β and IL-6 in the infarct border zone both of day 1 and day 7 after LAD ligation compared with CTL (Figure 4B).

**Figure 5:**
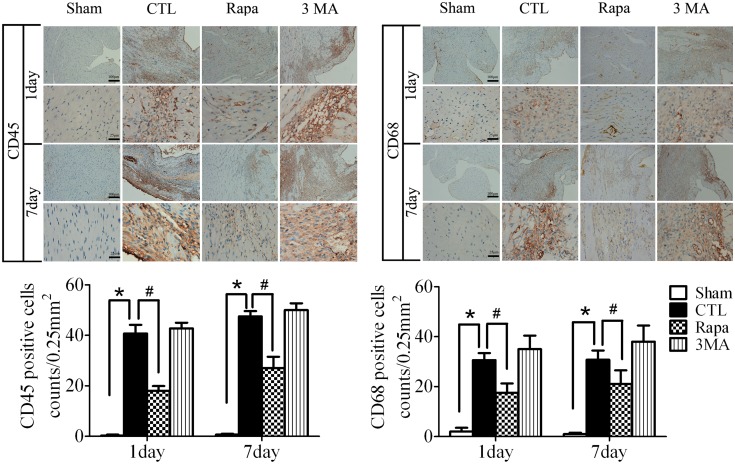
Rapamycin attenuated the inflammatory cell infiltration after LAD ligation. The representative images and statistical result of immunohistochemical assay of the infarct border zone of different time points after LAD ligation to test the infiltration of CD45^+^ leukocytes, including CD68^+^ macrophages (n = 5, *P<0.05 *vs* Sham; #*P*<0.05 *vs* CTL). Sham, mice without LAD ligation; CTL, LAD ligation with saline; Rapa, LAD ligation with Rapamycin treatment; 3MA, LAD ligation with 3MA treatment.

In addition, we examined the inflammatory cell infiltration in the infarct border zone after LAD ligation by immunohistochemical analysis of CD68^+^ and CD45^+^ cells. As shown in Figure 5, infiltration of CD45^+^ leukocytes, including CD68^+^ macrophages, in the infarct border zone both of day 1 and day 7 after LAD ligation was restrained by Rapamycin treatment(*P*<0.05, *vs* CTL, n = 5).

### Rapamycin inhibited NFκB activation induced by ischemia and inhibition of NFκB activated autophagy in a negative reflex

**Figure 6:**
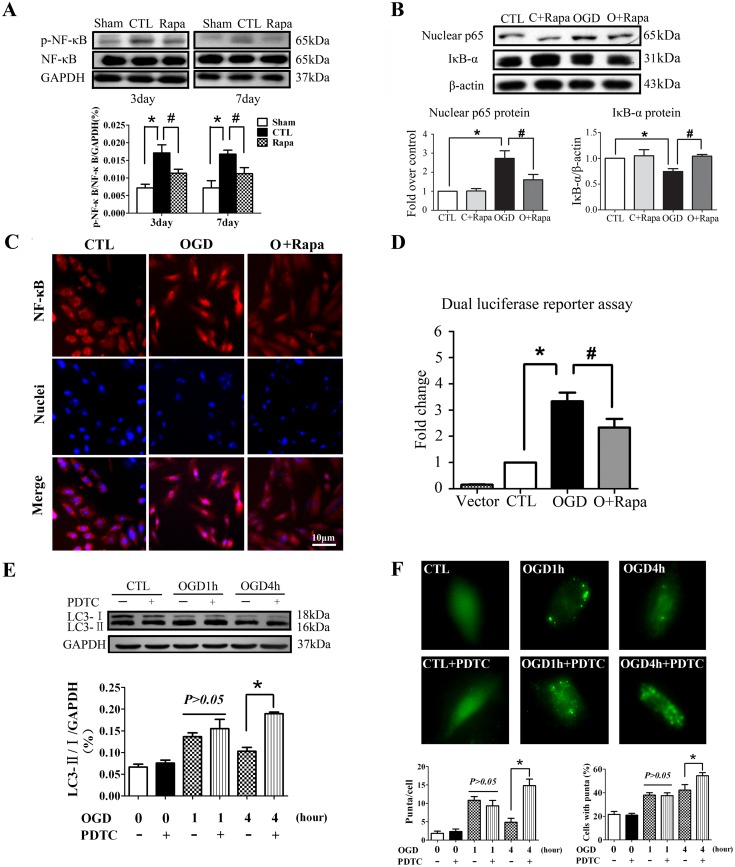
Rapamycin inhibited NFκB activation after myocardial ischemia and inhibition of NFκB activated autophagy in return. **A**, Rapamycin inhibited NFκB phosphorylation in the border zone of ischemic hearts tested by Western blotting (n = 5, *P<0.05 *vs* Sham; #*P*<0.05 *vs* CTL). **B**, The effect of Rapamycin on nuclear p65 and cytoplasm IκBα of the H9C2 cells after OGD tested by Western blotting (n = 5, *P<0.05 *vs* CTL; #*P*<0.05 *vs* OGD). **C**, The effect of Rapamycin on p65 translocation to the nuclei in the H9C2 cells after OGD tested by immunostaining assay. **D**, The effect of Rapamycin on the transcriptional activity of NFκB in the H9C2 cells after OGD (n = 6, *P<0.05 *vs* CTL; #*P*<0.05 *vs* OGD). **E and F**, inhibition of NFκB upregulated LC3 protein modification in the H9C2 cells 4 hours after oxygen glucose deprivation for by Western blot (E) and Immunostaining assay (F) (*P<0.05 *vs* OGD, n = 6) OGD, oxygen glucose deprivation; CTL, normal control with serum and oxygen; C+Rapa, normal control with Rapamycin treatment; O+Rapa, OGD with Rapamycin treatment.

NFκB is widely believed as a critical transcription factor which is closely associated with inflammation in AMI [20], [21]. *In vivo* we found that Rapamycin inhibited NFκB phosphorylation in the hearts of AMI (Figure 6A). *In vitro*, IκBα degradation was significantly induced after OGD for 1 hour, which was reversed by Rapamycin pretreatment. The nuclear p65 was increased in the H9C2 cells after OGD treatment, which was also reversed significantly by Rapamycin pretreatment (Figure 6B). Likewise, the results of immunostaining assay confirmed that Rapamycin inhibited the nuclear translocation of NFκB with OGD (Figure 6C). Moreover, we performed dual luciferase reporter assay to test the effect of Rapamycin on the transcriptional activity of NFκB and found that Rapamycin significantly reduced the NFκB transcription activity induced by OGD treatment in the H9C2 cells (Figure 6D). These data together indicated that the enhanced autophagy might inhibit NFκB activation after AMI.

To investigate whether the inhibition of NFκB affect the autophagy process, we treated the H9C2 cells with NFκB inhibitor, PDTC. The result showed that inhibition of NFκB activated autophagy in return. Western blot assay showed that LC3 protein modification was significantly increased with PDTC after OGD treatment for 4 hours (Figure 6E). The LC3 protein modification was confirmed by GFP-LC3 immunostaining assay. As shown in Figure 6F, PDTC pretreatment significantly increased GFP-LC3 positive cells and puncta in the H9C2 cells with OGD treatment for 4 hours. In addition, we tested the effect of PDTC on P62 degradation after OGD treatment. As shown in Figure S3, PDTC pretreatment significantly increased P62 degradation after OGD for 4 hours (*P*<0.05, O+PDTC *vs* OGD). Consequently, these results indicated that inhibition of NFκB activated autophagy in a negative reflex.

## Discussion

Our results showed that autophagy was induced dramatically at the acute stage of AMI, and however, impaired the subacute and chronic stage of AMI. Rapamycin, administered as post-treatment after myocardial infarction, enhanced autophagy and rendered the heart resistant to adverse cardiac remodeling of AMI. Of note, we found that Rapamycin attenuated the inflammatory response and NFκB activation provoked in AMI, and inhibition of NFκB restored autophagy in a negative reflex.

### The dynamics and functional role of autophagy in postinfarction cardiac remodeling

The dynamics and the functional role of autophagy during a longer period heart ischemia are not clear. In the present study, we observed the dynamics of autophagy in the infarct hearts for a longer period of 21 days after the LAD ligation. The results, confirmed by the protein marker expression and electron microscopy assay, showed that autophagy was augmented in the acute stage of AMI and then began to decrease 5 days after LAD ligation (Figure1). It should be noted that three principal methods, including electron microscopy, light microscopy detection of the subcellular localization of LC3, and biochemical detection of the membrane-associated LC3, are presently used to monitor autophagosomes. However, there will be an increase of autophagosome either in the situation of autophagy activation or decreased autophagic degradation [22],[23]. Therefore, simple determination of autophagosomes is insufficient for an overall estimation of the autophagic activity. Rather, autophagic flux by testing the autophagy substrate, P62, is preferred. P62 degradation in the infarct border zone was increased from day 1 to day 3 after LAD ligation, and then decreased from day 5 to day 21 after LAD ligation (Figure S1A), which helps to confirm that autophagy was stimulated in the acute ischemia phase, and however impaired in the latter phase of AMI. The *in vitro* study showed that P62 degradation was increased 2 hours after OGD, and however P62 was accumulated at 12 hours after OGD, which was consistent with the *in vivo* result. In addition, we measured P62 *in vitro* to assess the autophagy flux in the H9C2 cells under OGD with the lysosomal inhibitor Chloroquine. There was no difference of P62 between in the presence and absence of Chloroquine after OGD treatment indicating the autophagic flux was impaired after OGD treatment (Figure S2). Kanamori et al detected a sustained increase of LC3 dots in the remote area after AMI [9]. However, their results showed that LC3 dots was also decreased in the border area of the hearts of 2 and 3 weeks postinfarction and P62 degradation was blocked in the hearts of 1 or 3 weeks postinfarciton, which is consistent with our study. Lin Y et al showed that autophagy was upregulated in the chronically ischemic myocardium; however, they use the different model in which the pigs were subjected to repetitive myocardial ischemia produced by one, three, or six episodes of 90 minutes of coronary stenosis [24]. Our results showed autophagy was induced sharply after LAD ligation, and however, sustained myocardial ischemia impaired cardiomyocyte autophagy, indicating that autophagy is an early stress reaction which is compensatory. We supposed that the impaired autophagy in the latter phase of AMI may deprive the protection against ischemia and exacerbate cardiac remodeling.

To investigate the mechanism and significance of the impaired autophagy during the subacute and chronic stages of AMI, we treated the mice with Rapamycin one day after LAD ligation to make up the insufficient autophagy. Indeed, Rapamycin induced autophagy in the heart tissue in the mice and H9C2 cells. Treatment of Rapamycin significantly mitigated cardiac dysfunction and remodeling. Conversely, 3MA inhibited autophagy and significantly exacerbated cardiac dysfunction and remodeling. These results suggested that autophagy in the surviving cardiomyocyte is one of the compensatory mechanisms that attenuate postinfarction cardiac dysfunction and remodeling.

Actually, the dynamics of autophagy during a long period of myocardial ischemia remains controversial. We are not sure about the etiology or significance of the impaired autophagy during the subacute and chronic stages of AMI. However, it has been shown that pharmacological augmentation of autophagy protects against adverse remodeling of AMI injury by Kanamori et al [9], [14]. It makes more sense that autophagy was impaired in the subacute and chronic stage of AMI during which Rapamycin augment autophagy to make up this deficiency and protect against ischemia injury. Autophagy activation is complex and incompletely understood, leading to the activation of a wide range of signaling pathways [25], [26], [27], [28]. In our study, AMPK phosphorylation was induced both *in vivo* and *in vitro* (Figure S4). It was reported that autophagy induction following ischemia is AMPK dependent [7]. Hif-1α was also induced in the H9C2 cells after OGD for 2 hours (Figure S4). Further experiments are needed to explore the mechanism by which autophagy was induced in the acute phase and however impaired in the latter phase of AMI.

On the other hand, we investigated the effect of Rapamycin-enhanced autophagy on ischemic injury in H9C2 cells, which is widely-used *in vitro* model for investigation of heart hypoxia [29]. We failed to establish ideal ischemia model in the neonatal rat cardiomyocytes or the adult rat cardiomyocytes mainly because both of these cells exhibit good tolerance to hypoxia or oxygen glucose deprivation injury (data not shown). In line with the *in vivo* study, Rapamycin induced autophagy and protected the H9C2 cells from oxygen glucose deprivation injury. These results underlined that Rapamycin induced autophagy after AMI mimics a conserved process for survival of mammalian cells.

Of note, medication in this study was initiated one day after induction of AMI, thus closely resembling a frequent clinical situation. The mTOR inhibitor, Rapamycin has an inhibitory effect on protein synthesis via direct inhibition of mTOR. It has been used as proliferative inhibitors for coating drug–eluting stents to decrease restenosis after coronary angioplasty. Sirolimus is even effective to prevent restenosis when given orally for a couple of years [30]. For AMI, despite the widespread use of therapeutics intervention with the neurohumoral axis, the incidence of heart failure with the end-stage of LV remodeling remains high. In the present study, clinically relevant dose of Rapamycin attenuated cardiac dysfunction and adverse remodeling after myocardial infarction *in vivo* and *in vitro,* which provided evidence that the mTOR inhibitor Rapamycin could be used for preventing LV remodeling after AMI.

### The mechanism by which enhanced autophagy protects against adverse cardiac remodeling in AMI

Inflammation is an important process after AMI spreading of the infarct zone [31], [32]. It is found that diffuse and active inflammatory cells are infiltrated in both vulnerable and stable coronary plaques of patients dying of AMI [32], [33]. The production of IL-1β and IL-18 is increased in the absence of functional ATG16L1 in a mouse model of Crohn’s disease [34]. Several convergent reports showed that autophagy has a negative role in inflammasome activation [35], [36], [37]. Blockade of autophagy will result in an accumulation of depolarized mitochondria that leak endogenous inflammasome ago­nists, such as mitochondrial DNA [35], [36]. It was shown that mitochondrial DNA that escapes from autophagy cell-autonomously leads to Toll-like receptor 9-mediated inflammatory responses in cardiomyocytes and is capable of inducing myocarditis and dilated cardiomyopathy [38]. However, it is still unclear whether inflammation is involved in autophagy-induced protection against AMI. The enhanced autophagy attenuating inflammation in AMI was supported by our findings: 1) Rapamycin treatment decreased the inflammatory cytokines such as TNF-α, IL-1β and IL-6 (Figure 4) and decreased the inflammatory cells infiltration of leukocytes including macrophages (Figure 5). 2) Rapamycin treatment inhibited NFκB activation induced by AMI (Figure 6).

NFκB is an essential transcriptional factor in inflammation. It was reported that transfection of cis element decoy against NFκB or deletion of p50 subunit of NFκB reduced AMI infarct size [20], [21]. The ubiquitin proteasome system is required for NFκB activation and the proteasome inhibition blocks activation of NFκB after myocardial ischemia [39]. Our data showed that the enhanced autophagy suppressed the release of the inhibitory subunit IκBα from the latent cytoplasmic form of NFκB and inhibited NFκB activation. Consistent with our study, Qing G et al showed that autophagy reduced NFκB activation [40], [41]. IKB kinase (IKK), an essential activator of NFκB, is selectively degraded by autophagy, which provided an important insight into the mechanism by which autophagy inhibited NFκB activation by regulating IKK stability independent of proteasome [41]. NFκB signaling may be down-regulated by autophagy via NSFL1C cofactor p47, which is a protein that has an ubiquitin-binding UBA domain and its orthologue in yeast binds to ATG-8. This potential adaptor acts as a negative regulator of IKK through the lysosomal (and presum­ably autophagic) degradation of poly-ubiquitylated NFκB essential modulator (NEMO) [42]. In addition, our understanding of the molecular and cellular mechanisms of autophagy involved in inflammation and postinfarction cardiac remodeling is still in its infancy. It nevertheless extends the idea that the attenuated inflammation will affect myocardial autophagy. Our results showed that PDTC, the inhibitor of NFκB, induced LC3 protein modification and increased P62 degradation after OGD treatment, which indicated that NFκB inhibition activated autophagy in a negative reflex in some way.

In summary, our results supported the conclusion that Rapamycin, administered as a post-treatment after myocardial infarction, makes up the impaired autophagy in the latter phase of AMI and renders the mice heart resistant to the AMI injury. Of note, we found that Rapamycin attenuated the inflammatory response and NFκB activation provoked in AMI, and inhibition of NFκB restored autophagy in a negative reflex.

## Supporting Information

Figure S1 **Autophagy substrate P62 degradation in vivo and in vitro. A**, P62 in the infarct border zone of the different time points after LAD ligation was examined by Western blotting (n = 5, *P<0.05 *vs* Sham). **B**, P62 in the H9C2 cells for different time points after OGD (n = 5, *P<0.05 *vs* CTL).
(TIF)

Figure S2 **Effect of Chloroquine on P62 level in the H9C2 cells after oxygen glucose deprivation.** H9C2 cells were treated with or without Chloroquine (20 uM) for 2 hours and then subjected to OGD for 12 hours. There is no difference of P62 between in the presence and absence of Chloroquine after OGD treatment. (n = 5, *P<0.05 *vs* CTL).
(TIF)

Figure S3 **The effect of PDTC on P62 degradation in the H9C2 cells was examined by Western blotting.** The H9C2 cells were pretreated with PDTC for 1 hour and then subjected to OGD for 4 hours. (n = 3, *P<0.05 *vs* OGD).
(TIF)

Figure S4 **AMPK and HIF-1α were examined by Western blotting in vivo and in vitro.** A, AMPK expression in the infarct border zone of different time points after LAD ligation (n = 5, *P<0.05 *vs* Sham) B, AMPK and HIF-1α expression in the H9C2 cells after OGD treatment for different time. (n = 5, *P<0.05 *vs* CTL).
(TIF)
