# Supplementary figures and images for: Impaired Autophagy Contributes to Adverse Cardiac Remodeling in Acute Myocardial Infarction
Source: PLoS One. 2014 Nov 19;9(11):e112891. doi: 10.1371/journal.pone.0112891 (PMC4237367; doi:10.1371/journal.pone.0112891)

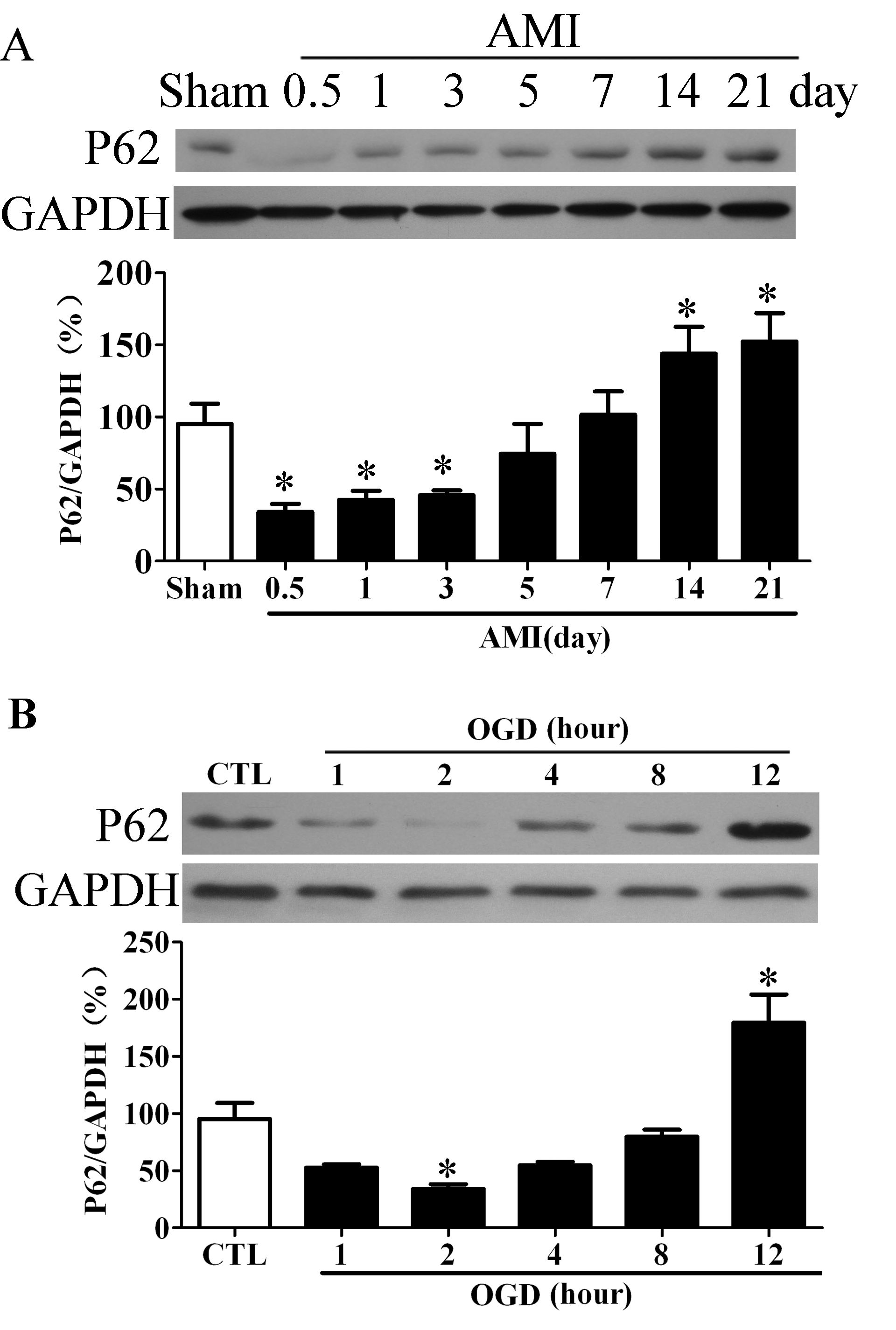

Supplement: Figure S1 — Autophagy substrate P62 degradation in vivo and in vitro. A, P62 in the infarct border zone of the different time points after LAD ligation was examined by Western blotting (n = 5, *P<0.05 vs Sham). B, P62 in the H9C2 cells for different time points after OGD (n = 5, *P<0.05 vs CTL). (TIF) [file pone.0112891.s001.tif]

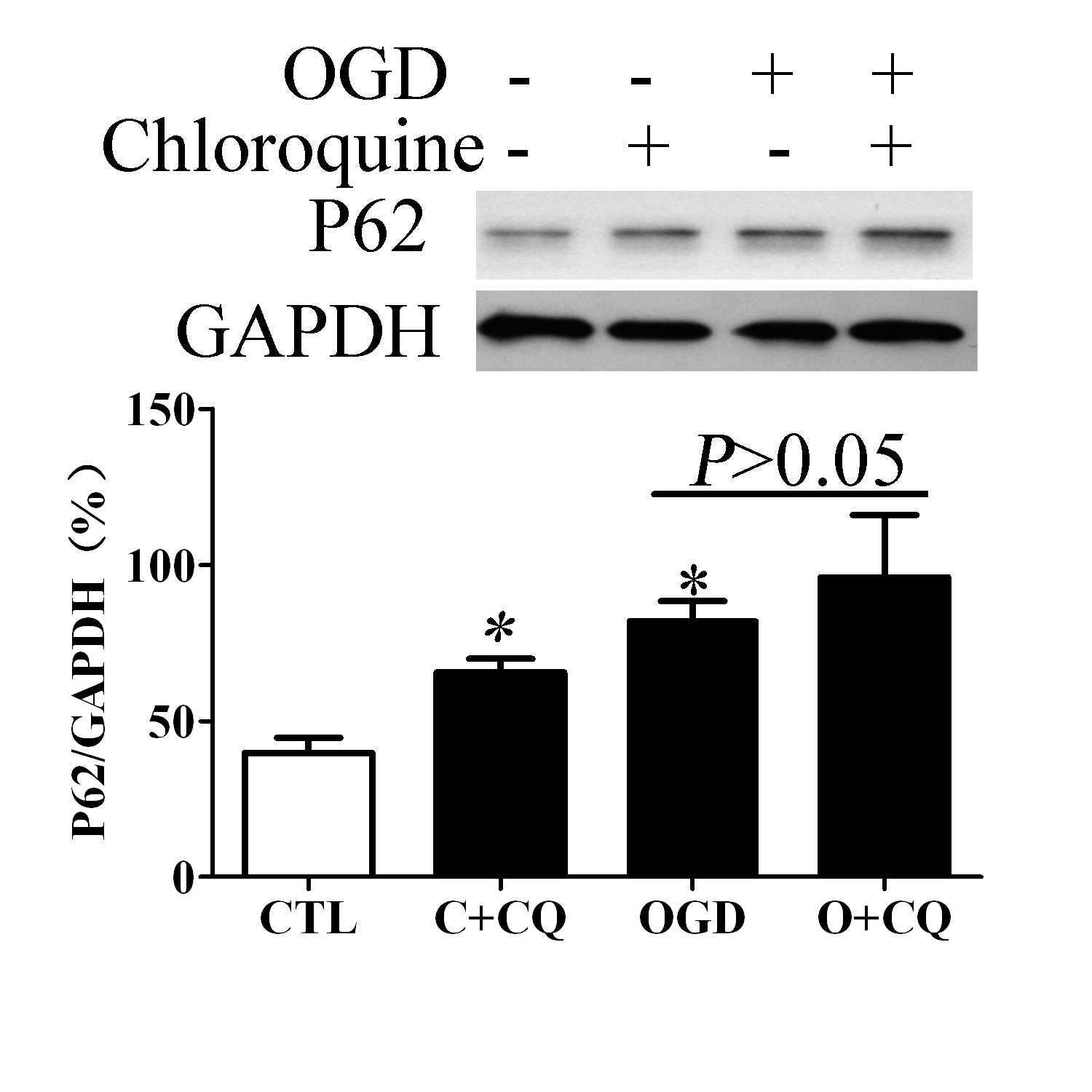

Supplement: Figure S2 — Effect of Chloroquine on P62 level in the H9C2 cells after oxygen glucose deprivation. H9C2 cells were treated with or without Chloroquine (20 uM) for 2 hours and then subjected to OGD for 12 hours. There is no difference of P62 between in the presence and absence of Chloroquine after OGD treatment. (n = 5, *P<0.05 vs CTL). (TIF) [file pone.0112891.s002.tif]

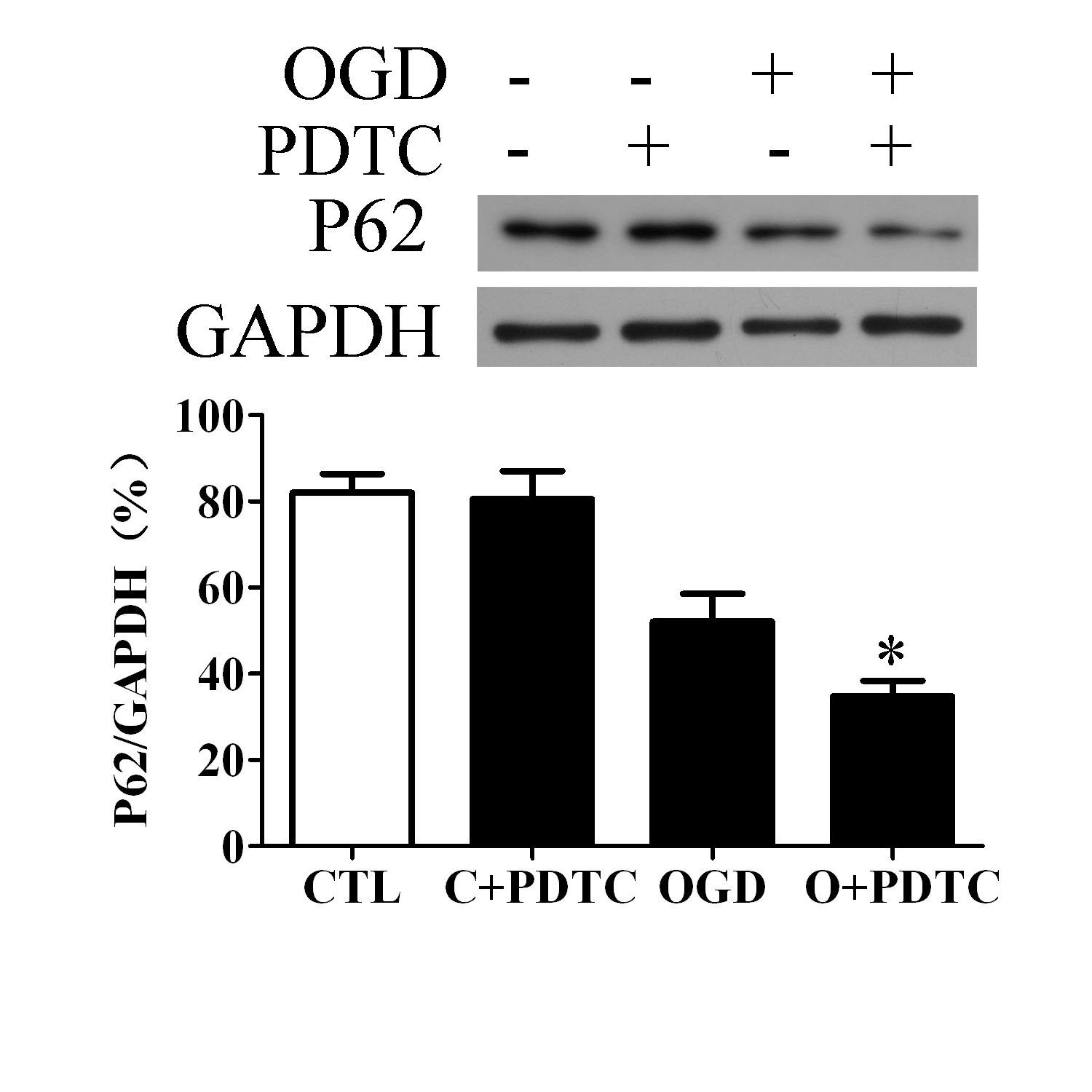

Supplement: Figure S3 — The effect of PDTC on P62 degradation in the H9C2 cells was examined by Western blotting. The H9C2 cells were pretreated with PDTC for 1 hour and then subjected to OGD for 4 hours. (n = 3, *P<0.05 vs OGD). (TIF) [file pone.0112891.s003.tif]

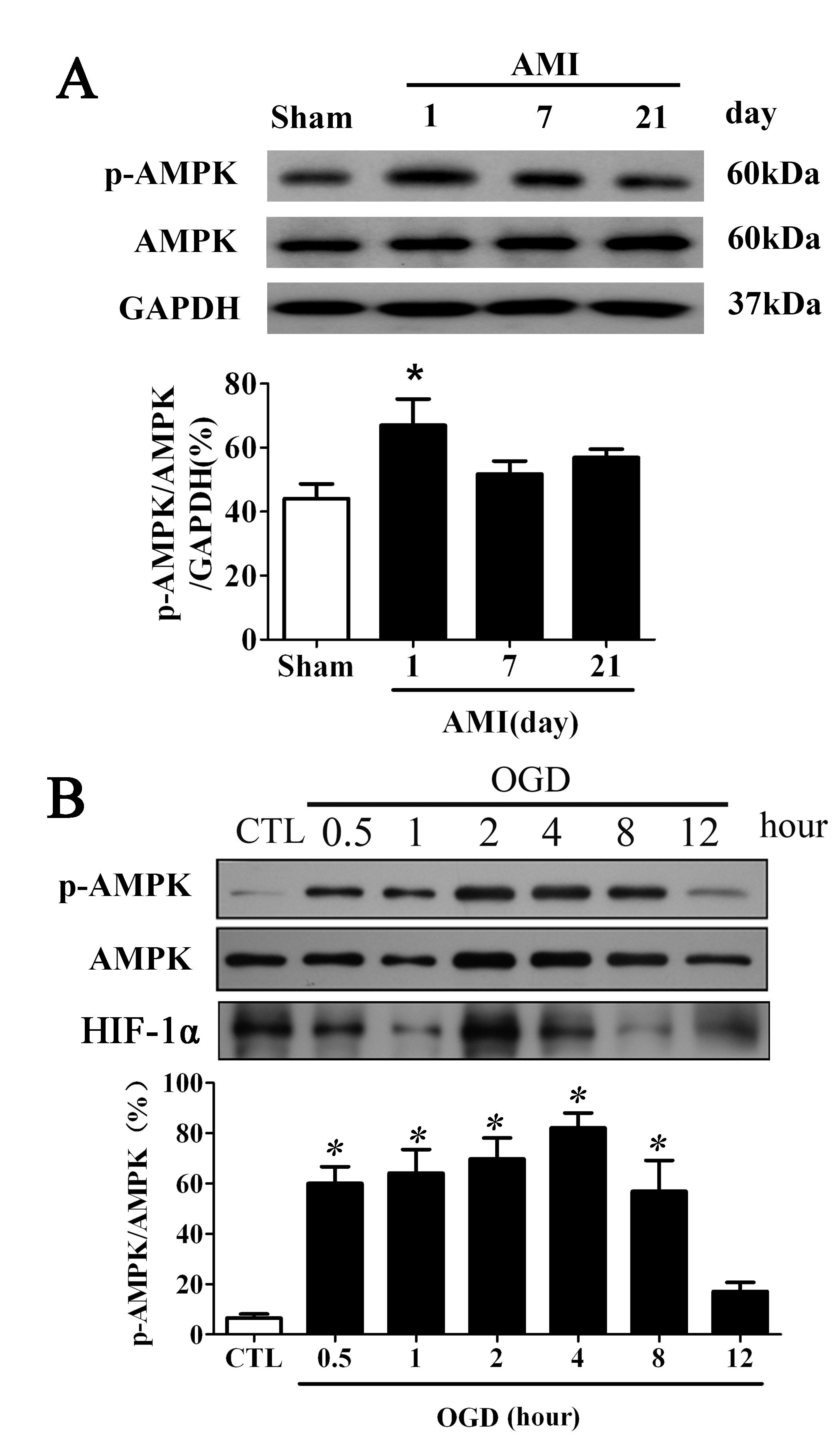

Supplement: Figure S4 — AMPK and HIF-1α were examined by Western blotting in vivo and in vitro. A, AMPK expression in the infarct border zone of different time points after LAD ligation (n = 5, *P<0.05 vs Sham) B, AMPK and HIF-1α expression in the H9C2 cells after OGD treatment for different time. (n = 5, *P<0.05 vs CTL). (TIF) [file pone.0112891.s004.tif]
